# Faecal calprotectin testing in UK general practice: a retrospective cohort study using The Health Improvement Network database

**DOI:** 10.3399/BJGP.2021.0125

**Published:** 2021-10-05

**Authors:** Karoline Freeman, Ronan Ryan, Nicholas Parsons, Sian Taylor-Phillips, Brian H Willis, Aileen Clarke

**Affiliations:** Warwick Medical School, University of Warwick, Coventry.; Institute of Applied Health Research, University of Birmingham, Birmingham.; Warwick Medical School, University of Warwick, Coventry.; Warwick Medical School, University of Warwick, Coventry.; Institute of Applied Health Research, University of Birmingham, Birmingham.; Warwick Medical School, University of Warwick, Coventry.

**Keywords:** diagnostic tests, general practice, inflammatory bowel diseases, practice guidelines as topic

## Abstract

**Background:**

Faecal calprotectin (FC) testing to detect inflammatory bowel disease (IBD) was recommended for use in UK general practice in 2013. The actual use of FC testing following the national recommendations is unknown.

**Aim:**

To characterise the use of FC testing for IBD in UK general practice.

**Design and setting:**

A retrospective cohort study of routine electronic patient records from The Health Improvement Network database from UK general practice.

**Method:**

The study included 6 965 853 adult patients (aged ≥18 years), between 2006 and 2016. FC test uptake, the patients tested, and patient management following testing were characterised.

**Results:**

A total of 17 027 patients had 19 840 FC tests recorded. The mean age of tested patients was 44.2 years. The first FC tests were documented in 2009. FC test use was still increasing in 2016. By 2016, 66.8% (*n* = 493/738) of practices had started FC testing. About one-fifth (20.7%, *n* = 1253/6051) of tests were carried out in patients aged ≥60 years. Only 7.8% (*n* = 473/6051) of the FC test records were preceded by symptoms eligible for FC testing. Only 3.1% (*n* = 1720/55 477) of patients with eligible symptoms have received FC testing since the national recommendations were published. There was only a small number of patients with symptoms, FC test, and a IBD diagnosis. In total, 71.3% (*n* = 1416/1987) of patients with a positive and 47.7% (*n* = 1337/2805) with a negative FC test were referred or further investigated.

**Conclusion:**

Uptake of FC testing in clinical practice has been slow and inconsistent. The indication of non-compliance with national recommendations may suggest that these recommendations lack applicability to the general practice context.

## INTRODUCTION

Faecal calprotectin (FC) is a biomarker for gastrointestinal inflammation. FC testing is recommended by gastroenterological societies across the globe for its usefulness in the diagnosis of inflammatory bowel disease (IBD), a chronic inflammatory condition requiring specialist treatment.^1^^–^5 However, there is no clear guidance on settings, in which it is considered appropriate.

In the UK, FC testing was approved by the National Institute for Health and Care Excellence (NICE) in 2013 for use in general practice in patients when referral to secondary care is being considered and cancer is not suspected (diagnostics guidance: DG11)^6^ to reduce the number of unnecessary referrals to colonoscopy. The NICE evaluation defined eligibility for testing as ongoing abdominal symptoms for ≥6 weeks in patients aged <45 years. The assessment also recommended referral of patients with FC levels ≥50 µg/g. At the time of publication of the guidance, little evidence of FC testing in general practice was available.

Insights into how the FC test may work in general practice came from two small pilot studies in the north of England funded by the NHS Technology Adoption Centre.^7^ The findings from these pilot studies mainly concerned the theoretical referral pattern of patients with abdominal symptoms based on test results but did not consider the impact of testing on decision making, as the working diagnosis and referral decision were not influenced by the FC test. The guidance was primarily based on evidence from secondary care and assumptions on test use. The evidence was considered by an independent committee (the Diagnostics Advisory Committee including two consultant gastroenterologists and two GPs) that prepared the recommendations for NICE.

A recent systematic review concluded that there is still a lack of evidence on the defined role of FC testing in the general practice pathway for the detection of IBD.^8^ Furthermore, early experience with FC testing in general practice reported that between 26% and 45% of patients with a negative FC test result were referred to specialist care,^9^^–^11 which may question the usefulness of FC tests in general practice.

The aim of this study was to investigate the uptake of FC testing into routine general practice following NICE guidance, to characterise FC test use in light of NICE guidance, and to describe the impact of FC testing on referral and colonoscopy decisions using routine electronic health records from UK general practices.

## METHOD

### Data source

Study data consisted of electronic primary healthcare data from The Health Improvement Network (THIN). THIN consists of anonymised, longitudinal individual-level patient data from >670 UK general practices using the Vision practice software. In 2015 a total of >14 million patients had contributed data to THIN, which reflects a coverage of about 6% of the UK population.^12^ Data were included into the study from general practices from the date that the practice was deemed to be reporting all-cause mortality reliably compared with national statistics and from 1 year after the installation of the electronic medical record system.^13^ For additional information on the database see Supplementary Appendix S1.

**Table 1:** How this fits in

Faecal calprotectin (FC) testing to detect inflammatory bowel disease (IBD) was recommended for use in UK general practice in 2013 to reduce referrals of individuals who do not have IBD. The recommendations were based on evidence from secondary care because evidence from general practice on test accuracy, test use, and the impact of testing on patient management was scarce. This study found that 20.7% of FC tests were in patients not considered eligible according to recommendations. In addition, only 3.1% of eligible patients have received FC testing since national recommendations were published, whereas nearly 50% of patients with a negative test result were referred. Current national recommendations may therefore lack applicability to the general practice context and need adjusting.

### Study design and study population

A retrospective cohort study was undertaken of patients who were aged ≥18 years during the period 1 January 2006 to 31 December 2016. The study cohort was open with patients entering and exiting at different times. Patients entered the study 1 year after they registered with the general practice or at age 18 years, whichever came later. Patients exited the study at the earliest of the following dates: deregistration with the practice; death; or 1 January 2017.

### Identifying IBD diagnosis, eligible symptoms, and investigations

IBD diagnoses were identified using the first recorded clinical code for IBD or first prescription of IBD-specific medication in the patient record (see Supplementary Table S1 for included drugs and Supplementary Table S2 for complete code lists). The definition of IBD included ulcerative colitis, Crohn’s disease, indeterminate colitis, and microscopic colitis to reflect the general practice context before confirmatory testing.

Symptoms of interest were abdominal complaints that would justify FC testing (change in bowel habit, diarrhoea, constipation, bloating, and abdominal pain). NICE eligibility was defined as a relevant recorded abdominal symptom followed by a second recording of a relevant symptom after ≥6 weeks and <3 months. This definition was used to operationalise NICE guidance, which stipulate eligibility for testing as chronic abdominal symptoms lasting ≥6 weeks.^6^ Clinical code lists were adapted from those used in previous literature where available.^14^

All coded records of FC tests were considered. Colonoscopies were included if they were recorded within 12 months of the FC test date. Therapeutic investigations and screening colonoscopies were excluded. Endoscopy was only included if the record specified that it was for lower gastrointestinal tract investigations. Referral was defined as referral to any specialty or any record of a discharge letter, out-patient appointment, or admission to hospital within 6 weeks of the index FC test. For counts of referral and colonoscopy, an IBD record was considered referral with colonoscopy positive under the assumption that an IBD diagnosis is not usually made without a confirmatory investigation at a secondary care setting.

### Analysis

#### FC test uptake and characterisation of patients with a FC test

FC test uptake was determined as the total number of patients who had an FC test per 1000 population including all FC tests recorded between 2006 and 2016. Uptake by practice was determined as a) first-ever FC test per practice for each practice over time; and b) the number of FC tests ordered per 1000 practice population by the number of practices in THIN between 2006 and 2016. FC tests were characterised in terms of the patients’ demographics and recorded symptoms within the 1 year before testing.

#### Relationship between patients with an FC test, IBD, and eligible symptoms

The number of patients with eligible symptoms for testing, with an FC test, with an IBD diagnosis, and with any combination of those three outcomes between 2013 (the publication year of FC guidance) and 2016 have been summarised with the aid of a Venn diagram using the online tool biovenn.^15^ First-ever recorded events with dates that followed the logical sequence: symptoms preceding FC testing preceding IBD diagnosis, using mutually exclusive categories, were used for this.

IBD prevalence with 95% confidence intervals (CIs) in patients who underwent FC testing was determined for 2013 to 2016 using age cut-offs between 40 and 60 years at the time of testing.

#### Referral and colonoscopy following FC testing

Proportions of referrals within 6 weeks and colonoscopies within 1 year of testing by FC test outcome (FC cutoff 50 µg/g) were determined. Only first-ever recorded FC tests without prior IBD diagnosis between 2009 and 2016 were included. FC tests without numeric results in µg/g were excluded from the analysis. The proportion of IBD diagnoses in patients who were referred and had colonoscopies were summarised by FC test result. Patients were only included if the complete follow-up time was available after testing so as not to introduce bias against late referrals/colonoscopies in individuals who tested negative compared with those who tested positive.

All analyses were undertaken in R (version 3.6.1) unless otherwise specified. Graphs were drawn using the package ‘ggplot2’.^16^

## RESULTS

### FC test uptake

**Figure 1.**
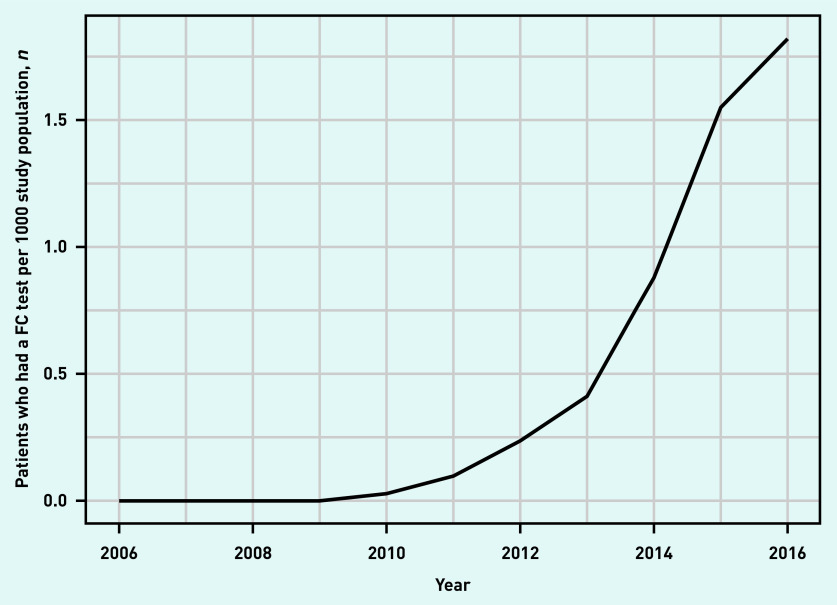
*Patients who had a FC test per 1000 study population by year. FC = faecal calprotectin.*

The study population consisted of 6 965 853 adult patients between 2006 and 2016. Of these, 17 027 patients (mean age 44.2 years) had a total of 19 840 FC tests recorded. There were 1820 patients who had two FC tests and 400 patients who had >2 tests. The number of tests per patient in the study period ranged from 1 to 13. Figure 1 depicts patients who had a FC test per 1000 population over time. The first FC tests were documented in 2009 with a noticeable increase in FC test use in 2013. FC test use was still increasing at the end of the study period.

**Figure 2.**
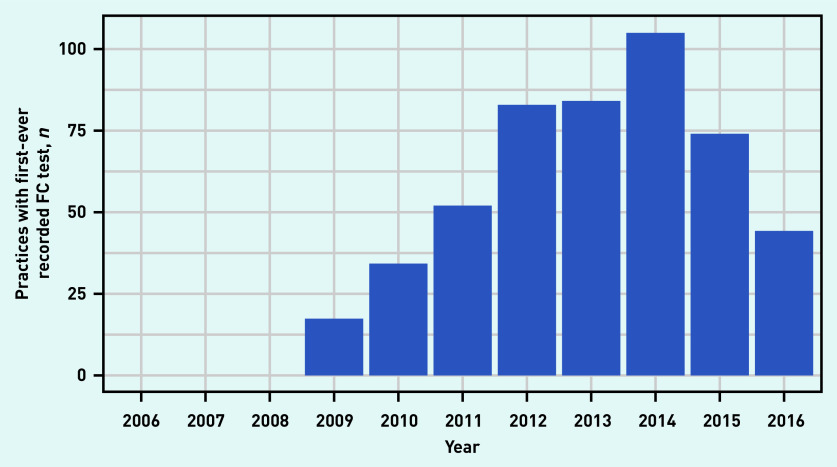
*Number of practices with first-ever recorded FC test between 2006 and 2016. FC = faecal calprotectin.*

**Figure 3.**
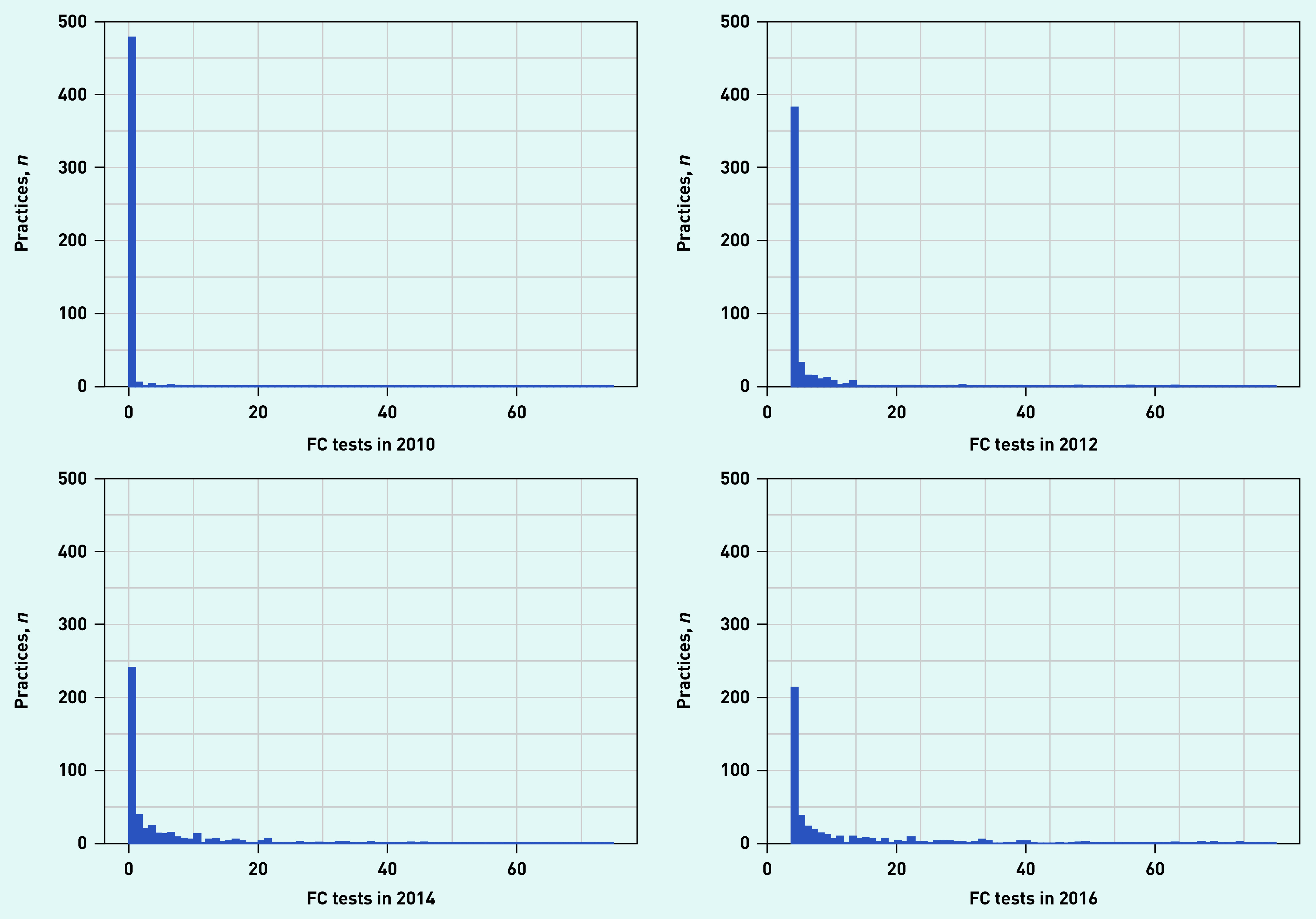
*Number of FC tests by number of practices for 2010, 2012, 2014, and 2016. FC = faecal calprotectin.*

Uptake by practice revealed that the number of practices recording their first FC test increased steeply between 2009 and 2012, and peaked in 2014 (Figure 2). By 2016, 66.8% (*n* = 493/738) of general practices had started using FC testing. The majority of practices used <5 FC tests in a single year throughout the study period (Figure 3). By 2016 the number of tests per practice ranged from 0 to >70.

Supplementary Table S3 provides a summary of the demographics and clinical characteristics of patients who had a FC test from 2009 to 2016. About one-fifth of tests (20.7%, *n* = 1253/6051) were in patients aged ≥60 years. Pain and diarrhoea were the most commonly coded abdominal symptoms within the 1 year before FC testing. In 2016, just over half of the tested patients had any abdominal symptoms recorded before testing. Of note, only 7.8% (*n* = 473/6051) of FC test records were preceded by a record of symptoms eligible for FC testing according to the NICE recommendations.^6^

### Patients with a FC test, IBD, and eligible symptoms between 2013 and 2016

**Figure 4.**
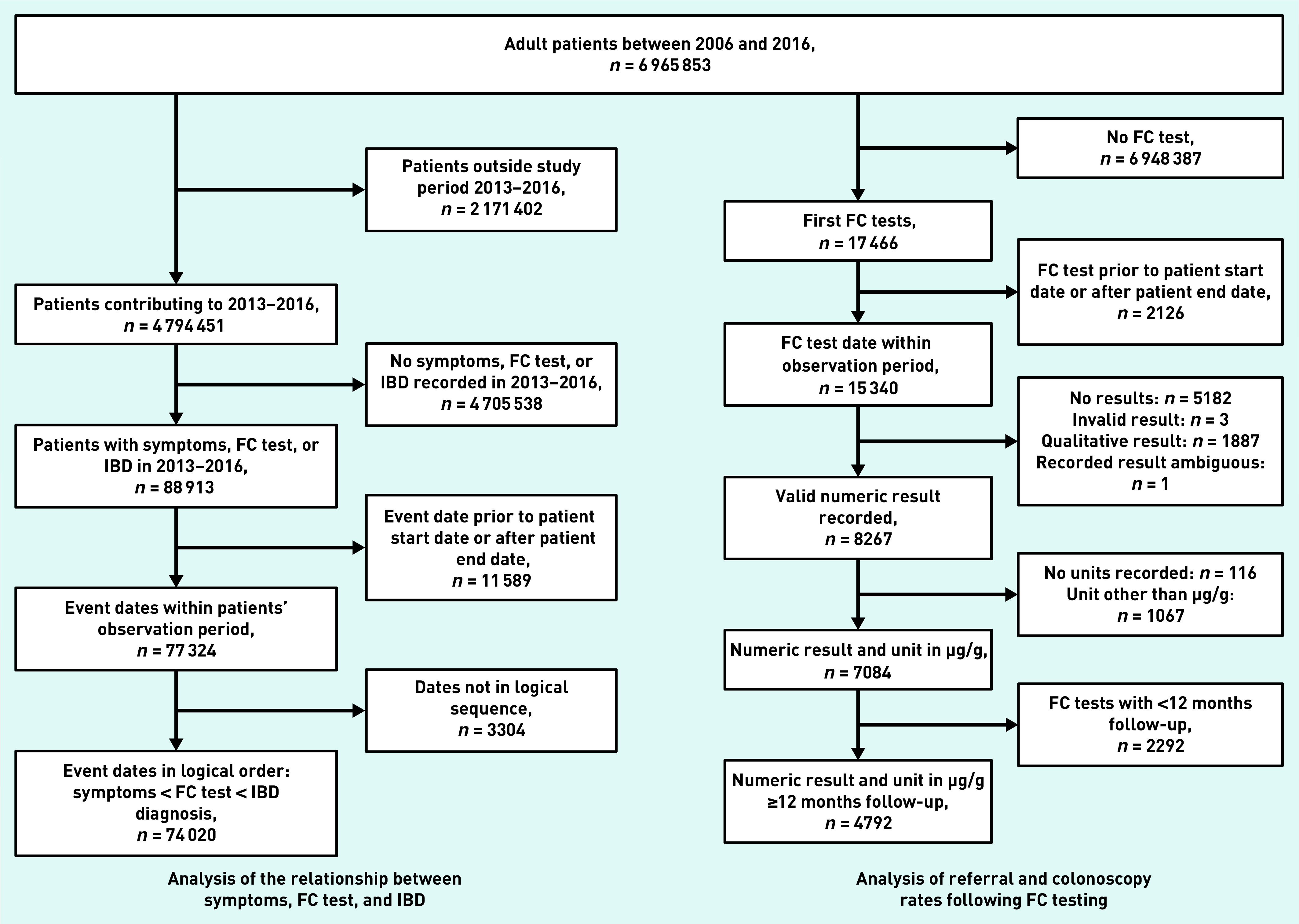
*Overview of inclusion and exclusion of patients for the two analyses. The relationship between symptoms, FC test, and IBD diagnosis (left) and referral and colonoscopy following FC testing (right) are shown. IBD = inflammatory bowel disease. FC = faecal calprotectin.*

Of 6 965 853 adult patients, there were 55 477 who had eligible symptoms for FC testing, 13 877 with an FC test record, and 7640 with an IBD diagnosis between 2013 and 2016. A total of 2974 patients had a combination of these events leading to a total of 74 020 patients with ≥1 of these events (Figure 4).

**Figure 5.**
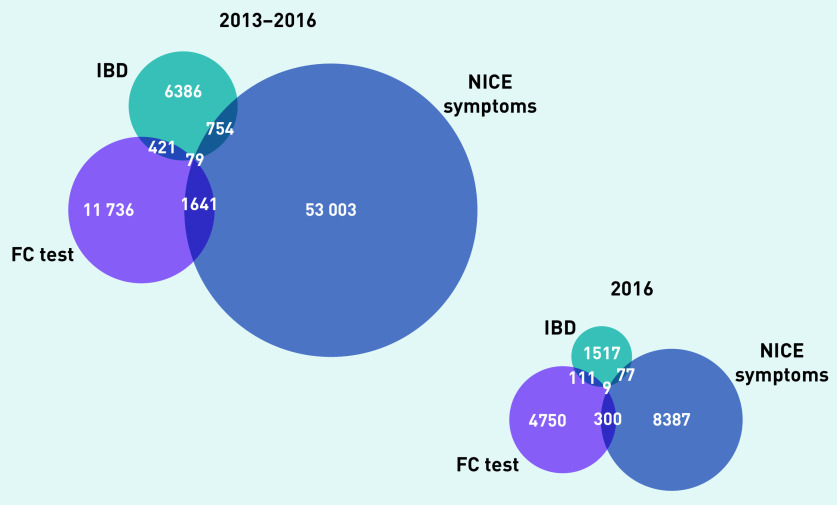
*Numbers of patients with eligible symptoms for FC testing, a record of FC testing, and/or an IBD diagnosis for 2013–2016 and for 2016 only. IBD = inflammatory bowel disease. FC = faecal calprotectin. NICE = National Institute for Health and Care Excellence.*

The Venn diagram in Figure 5 shows the proportional relationship of the three events in the population between 2013 and 2016. The number of patients with eligible symptoms followed by an FC test and an IBD diagnosis was small (*n* = 79/74 020).

Only 3.1% (*n* = 1720/55 477) of patients with eligible symptoms received an FC test. Furthermore, only 6.5% (*n* = 500/7640) of patients with an IBD diagnosis had a prior FC test. The proportion of FC tests ordered in patients without eligible symptoms and without subsequent IBD diagnosis was much larger. Restricting the analysis to the year 2016 (including a total of 15 151 patients) did not show a change in the pattern (Figure 5).

The IBD prevalence in 13 877 patients who had a FC test was 3.4% (95% CI = 3.1% to 3.7%) (see Supplementary Table S4). Adding age as an eligibility criterion showed that prevalence was similar using age cut-offs from 40 to 60 years, with point estimates slightly decreasing. In patients who had a FC test, 27.8% (*n* = 130/468) of IBD diagnoses were in patients aged ≥50 years.

### Referral and colonoscopy following FC testing

**Figure 6.**
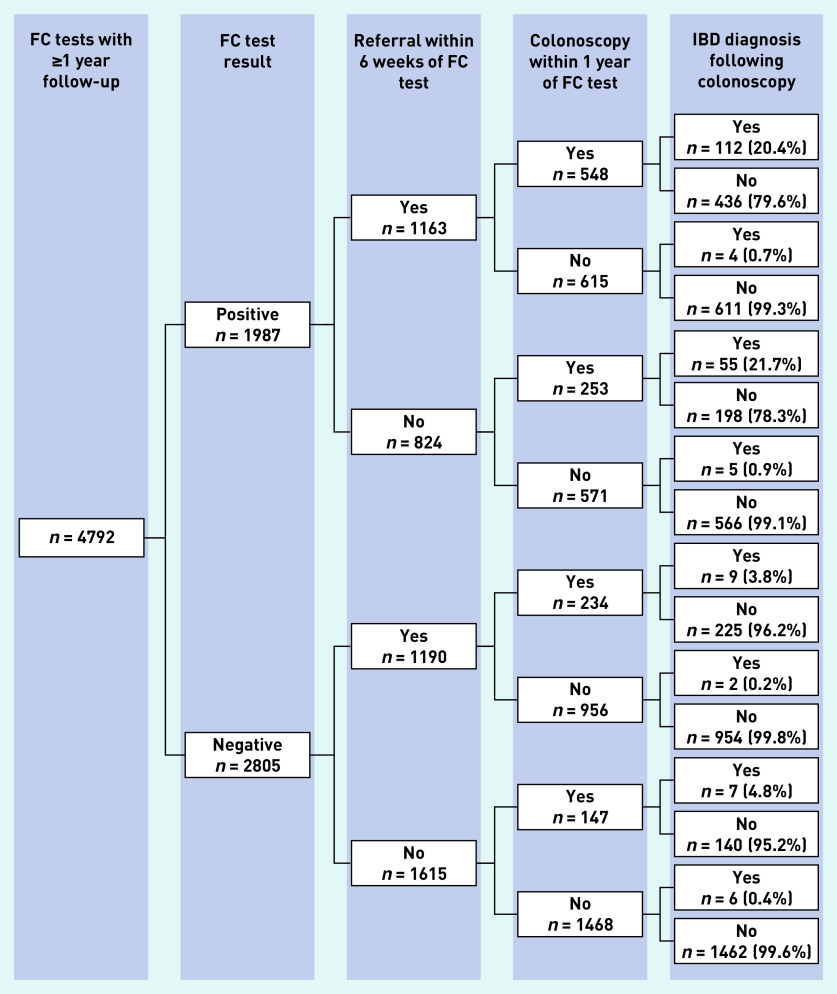
*Proportion of FC tests with referral, colonoscopy, and IBD diagnosis by FC test outcome (FC cut-off 50 µg/g). IBD = inflammatory bowel disease. FC = faecal calprotectin.*

Of 6 965 853 adult patients, 7084 (0.1%) had a first FC test recorded in THIN between 2009 and 2016. Of these, 4792 (67.6%) had at least 12 months follow-up (Figure 4). Figure 6 shows the proportion of IBD diagnoses in patients who were referred and those who received a colonoscopy by FC test result for the 4792 patients who had a FC test. The main finding was that nearly 50% (*n* = 1337/2805, 47.7%) of patients with a negative FC test were referred and/or received a colonoscopy in the defined time periods (compared to 71.3% [ *n* = 1416/1987] of patients with a positive FC test), with a small yield of IBD diagnoses.

## DISCUSSION

### Summary

Uptake of FC testing into general practice has been slow. Four years after the publication of the national recommendations on FC, only 66.8% of general practices had started using FC testing and the frequency of testing varied widely across practices. About one-fifth (20.7%) of tests were carried out in patients aged ≥60 years in whom testing has not been recommended. Few patients followed the anticipated NICE pathway (symptoms < FC testing < IBD diagnosis) and GPs appeared not to manage patients as indicated by the FC test results. Nearly 50% of patients with a negative FC test were referred and/or received a colonoscopy. Study findings indicate non-compliance with NICE guidance, which suggests insufficient information for GPs on who to test and how to act on the test result.

### Strengths and limitations

THIN is a rich source of routine electronic patient records of patients managed in general practice and is particularly useful in the study of real-world problems.^12^^,^^17^ The study population covered 6% of the UK population meaning that findings are expected to be generalisable to UK general practice.^12^ The reported prevalence of 3.4% of IBD in patients who had a FC test is broadly in line with published figures from FC test accuracy studies in UK primary care studies,^9^^–^11^,^^18^^–^20 and higher than the figures for the adult general practice population (1.4%) in 2016 in the same dataset.^21^

Missing data may have affected the current analysis. Secondary care investigations and specialist referrals are incomplete in general practice records. The extent of potential missed IBD diagnoses and symptoms because of incorrect/missed coding or free-text records in this study is not known. Variables to mitigate against potential missing data were defined.

FC test results were missing in 30% of recorded FC tests. Imputation methods were considered inappropriate because the proportion of missing data was too large, data were unlikely to be missing at random, and data essential for imputation (symptom severity, reason for testing, assay type, and/ or referral to gastroenterology) were not available.

Operationalising the NICE guidance on the eligibility of FC testing was difficult. In this study chronic symptoms were defined as having two instances of a clinical code for any gastrointestinal symptom recorded ≥6 weeks but <3 months apart. This would have missed patients with a free-text note concerning ongoing symptoms. The authors remain cautious in the interpretation of eligibility.

The end date of this study cohort was 31 December 2016. Uptake may have improved, and GPs may have got better at testing the right patients over the past 4 years. However, the authors do not know by how much. The data in this study did not show a change in pattern of FC test use from 2013 to 2016, and the authors are not aware of any major information drive to get GPs to use more FC testing and use it more appropriately.

### Comparison with existing literature

Adoption of new technologies is supported by providing information on the technology’s consequences, advantages, and disadvantages rather than just information about the technology itself.^22^ The NICE guidance insufficiently provides this level of information.

NICE guidance recommends testing to distinguish IBD from irritable bowel syndrome when cancer is not suspected.^6^ This narrow population does not reflect the broader spectrum of disease found in general practice. The variation in numbers of FC tests that practices ordered per 1000 patients may suggest that GPs have different thresholds and different reasons for testing.

The NICE evaluation of FC testing recommends FC testing for patients aged <45 years.^23^ However, 20.7% of tests were carried out in patients aged ≥60 years. Furthermore, 27.8% of IBD diagnoses in patients who had a FC test were in patients aged ≥50 years. This goes in hand with the second reported peak of Crohn’s disease onset in those aged between 50 and 60 years.^24^ This may support the revised cut-off of 60 years for FC testing suggested for general practice.^25^ However, the new age cut-off may be in disagreement with the guidance on FC test use when cancer is not suspected and places the responsibility with the GPs to judge whether FC testing is appropriate, which requires fundamental knowledge on what the FC test measures and how to use it.

NICE guidance recommends referral of patients with FC levels ≥50 µg/g. FC testing has a high negative predictive value of 99.6% (95% CI = 99.3% to 99.7%) at this threshold (and a positive predictive value of 8.1%, 95% CI = 7.1% to 9.2%).^26^ Nevertheless, the present study showed that nearly 50% of patients with an FC level <50 µg/g were referred into secondary care services. This is in agreement with reported estimates from several primary care studies.^9^^–^11^,^^18^^,^^19^

The referral of patients who are FC negative raises concerns over the impact of FC testing on colonoscopy rates as a considerable proportion of patients were further investigated. However, the number of colonoscopies in referred patients with FC negative tests varied greatly among the present study (13.6%, *n* = 381/2805) and three published studies (19%,^11^ 46%,^10^ and 71%^9^). This suggests that the number of potentially unnecessary colonoscopies may be at least as dependent on secondary care decision making as on the availability of FC testing in general practice, and that FC testing in primary care does not directly influence the decisions by specialists over whether to investigate referred patients. This present study cannot explain the reasons for referring patients with low FC levels in whom IBD is unlikely. It suggests that GPs have a lack of trust in a FC test result or have other reasons for referral, which FC testing may not be able to address.

### Implications for practice

The NICE guidance on FC testing may not be sufficiently informative and appropriate to promote uptake or consistent use of FC testing. This may be because, first, guidelines describe the test pathway for IBD rather than for abdominal pain using a diagnostic rather than a symptom-based approach. Second, guidelines are not sufficiently prescriptive to support a non-referral. Third, the evidence underlying the NICE guidance is not based on the setting in which the test is used. Therefore, the guidance is unable to provide the evidence that reducing the number of referrals to colonoscopy can be done at the same quality and safety of care. This may have led to noncompliance with NICE recommendations. The resulting lack in the expected reduction in colonoscopies may question the cost-effectiveness of FC testing as it is currently used in general practice.

NICE guidance may need revising and updating to make guidance applicable to general practice, and to provide more information for GPs on test use and test interpretation. NICE may need to change its approach in formulating guidance for testing in general practice. An understanding about how a test is used and interpreted in general practice is needed to formulate applicable guidance.

## Data

The datasets generated and/or analysed during the current study are not publicly available under the data sharing agreement with the University of Birmingham on behalf of THIN.
